# Analysis of quadruplex propensity of aptamer sequences

**DOI:** 10.1093/nar/gkaf424

**Published:** 2025-05-16

**Authors:** Anne Cucchiarini, Michaela Dobrovolná, Václav Brázda, Jean-Louis Mergny

**Affiliations:** Laboratoire d’Optique et Biosciences, Ecole Polytechnique, CNRS, INSERM, Institut Polytechnique de Paris, 91120 Palaiseau, France; Institute of Biophysics, Czech Academy of Sciences, Královopolská 135, 61200 Brno, Czech Republic; Faculty of Chemistry, Brno University of Technology, Purkyňova 118, 61200 Brno, Czech Republic; Institute of Biophysics, Czech Academy of Sciences, Královopolská 135, 61200 Brno, Czech Republic; Faculty of Chemistry, Brno University of Technology, Purkyňova 118, 61200 Brno, Czech Republic; Laboratoire d’Optique et Biosciences, Ecole Polytechnique, CNRS, INSERM, Institut Polytechnique de Paris, 91120 Palaiseau, France; Institute of Biophysics, Czech Academy of Sciences, Královopolská 135, 61200 Brno, Czech Republic

## Abstract

Aptamers are short DNA or RNA sequences that can fold into unique three-dimensional structures, enabling them to bind specifically to target molecules with high affinity, similar to antibodies. A distinctive feature of many aptamers is their ability to adopt a G-quadruplex (G4) fold, a four-stranded structure formed by guanine-rich sequences. While G4 formation has been proposed or demonstrated for some aptamers, we aimed to investigate how frequently quadruplex-prone motifs emerge from the SELEX process. To achieve this, we examined quadruplex candidate sequences from the UTexas Aptamer Database, which contains over 1400 aptamer sequences extracted from 400 publications spanning several decades. We analyzed the G4 and i-motif propensity of these sequences. While no likely i-motif forming candidates were found, nearly 1/4 of DNA aptamers and 1/6 of RNA aptamers were predicted to form G4 structures. Interestingly, many motifs capable of forming G4 structures were not previously reported or suspected. Out of 311 sequences containing a potential stable G4 motif, only 53 of them (17%) reported the word “quadruplex” in the corresponding article. We experimentally tested G4 formation for 30 aptamer sequences and were able to confirm G4 formation for all the sequences with a G4Hunter score of 1.31 or more. These observations suggest the need to reevaluate G4 propensity among aptamer sequences.

## Graphical Abstract

**Graphical Abstract:**
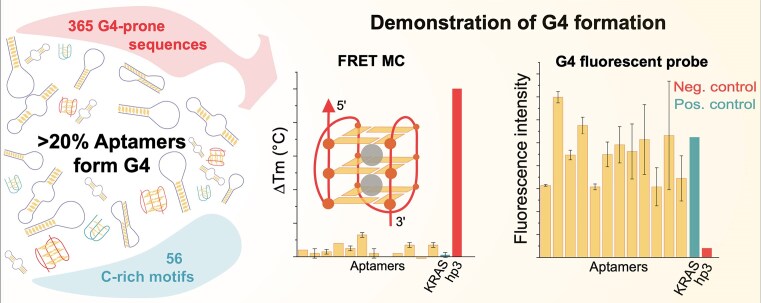

## Introduction

Aptamers are short, single-stranded DNA or RNA sequences that can fold into unique three-dimensional shapes. These structures enable aptamers to bind specifically to target molecules with high affinity, in a manner similar to antibodies [1, 2]. These targets range from small ions, biomolecules such as proteins or carbohydrates to whole cells. Aptamers are selected from vast nucleic acid libraries through an *in vitro* process known as SELEX (for Systematic Evolution of Ligands by Exponential Enrichment [3]). This method involves iterative rounds of binding, separation, and amplification to enrich sequences that have the highest affinity and selectivity for the target. This selection is made possible by starting with a DNA library containing a randomized region of variable length, flanked by sequences allowing amplification and, in the case of RNA aptamers, transcription. Unlike antibodies, aptamers are synthesized chemically, allowing for precise control over their composition, which leads to greater stability and reproducibility in diverse environments. In addition, multiple chemical modifications are available to stabilize their interaction or protect them from degradation, a general property of oligonucleotide therapeutics [4]. They can be easily modified to enhance their stability against nucleases, which is particularly advantageous for *in vivo* applications. Additionally, aptamers can be conjugated with various molecules, such as fluorescent dyes or nanoparticles, to enhance their detection capabilities. Their ability to bind to a wide range of targets, from small molecules to proteins and even whole cells, underscores their versatility and potential in scientific and medical fields. During the last three decades, there has been an “exponential growth of aptamer publications, with roughly 19 000 aptamer papers indexed in Pubmed in 2023” according to [5].

As research progresses, the development of novel aptamers continues to expand, opening new avenues for their application in precision medicine, environmental monitoring, and beyond. The high specificity and affinity of aptamers for their targets make them valuable tools in various applications. In diagnostics, aptamers can be used as biosensors to detect the presence of specific molecules, such as biomarkers for diseases [6, 7]. As biosensors [8], aptamers may be used to detect environmental contaminants, pathogens, or toxins. An example is the development of aptamer-based assays for the detection of bacteria such as Salmonella [9] and Listeria [10] in food safety testing, providing a rapid and sensitive means of ensuring food quality (for a review on aptamer biosensors for bacterial detection: [8]). In therapeutics, aptamers offer a promising alternative to traditional drugs due to their ability to bind precisely to disease-associated proteins and inhibit their function [11]. The first therapeutic aptamer was approved three decades ago. Macugen, an anti-vascular endothelial growth factor (VEGF) drug previously known as pegaptanib, was hailed as a revolutionary treatment for age-related macular degeneration (AMD), which had no other FDA-approved treatment at that time. It acts by preventing abnormal blood vessel growth in the eye [12]. A second aptamer therapeutic, Avacincaptad pegol (Izervay), was approved in August 2023 for the treatment of AMD [13]. Numerous others are in preclinical or clinical development.

A unique structural feature of some aptamers is their ability to adopt a G-quadruplex (G4) fold. G4s are four-stranded structures formed by DNA or RNA sequences rich in guanine (for review: [14]). These structures are stabilized by the stacking of two or more guanine quartets, which are square planar arrangements of four guanine bases connected through Hoogsteen hydrogen bonds. The formation of G4s is facilitated by the presence of monovalent cations such as potassium or sodium, which fit into the central channel of the quadruplex and stabilize the structure [15, 16]. Aptamers that adopt G4 folds can exhibit enhanced stability and binding specificity, making them suitable for targeting specific molecules. In contrast, the i-motif is an unusual DNA four-stranded structure formed by cytosine-rich sequences. The i-motif is composed of intercalated cytosine base pairs (C:C^+^), which are held together by hemi-protonation and Hoogsteen base pairing. The structural stability of i-motif DNA relies on the intercalative geometry of consecutive base pairs. Electrostatic interactions also play a crucial role in stabilizing the C:C^+^ base pairs. The study of i-motifs continues to grow, in particular for their use in nanotechnology as pH-sensitive biosensors andnanomachines.

G4s have been shown to play a critical role in the binding affinity and specificity of certain aptamers. There are actually several *thousand* hits in the Protein Data Bank (PDB) (https://www.rcsb.org) when searching it with the keywords “aptamer” and “quadruplex.” For example, an aptamer-based sensor has been developed for the detection of thrombin, a key enzyme in blood clotting, which can be crucial in monitoring blood coagulation disorders. The thrombin-binding aptamer (TBA), one of the most explored DNA aptamers, forms a well-characterized intramolecular DNA G4 structure composed of two quartets [17], allowing it to bind selectively to thrombin with high affinity. Another example is the AS1411 aptamer, which forms a G4 and targets nucleolin, a protein overexpressed in cancer cells [18]. The ability of aptamers to form unusual nucleic acid structures adds an additional layer of versatility to their application. This is especially the case for DNA G4s, which are highly polymorphic and can therefore adopt a variety of 3D shapes. The unique topological features of G4s provide specific binding sites that can be exploited for the selective targeting of molecules, enhancing the efficacy and specificity of aptamer-based therapeutics and diagnostics. In addition, G4s tend to be naturally resistant to nucleases, therefore enhancing the stability of aptamers in biological fluids and half-life *invivo*.

As research into G4-forming aptamers advances, we wanted to investigate how frequently quadruplex-prone motifs (G4 or i-motif) emerge from the SELEX process. To achieve this, we examined quadruplex candidate sequences found in the UTexas Aptamer Database, which is currently the largest repository of aptamer sequences [5]. This curated database includes over 1400 aptamer sequences extracted from 400 publications spanning several decades (1990–2023). We therefore analyzed the G4 and i-motif propensity of these 1400 sequences. Interestingly, while G4 formation has already been proposed or demonstrated for several aptamers, we identified motifs for which G4 formation had not been reported or suggested. Nearly 1/4 of DNA aptamers and 1/6 of RNA aptamers were predicted to form G4 structures, and we experimentally confirmed G4 formation for some aptamers that were not previously suspected of doing so.

## Materials and methods

### Selection of sequences

Aptamer sequences from the UTexas Aptamer Database (https://doi.org/10.1093/nar/gkad959) (https://sites.utexas.edu/aptamerdatabase/) were analyzed using G4Hunter (http://bioinformatics.ibp.cz) to identify sequences with the potential to form G4s.

Some of the aptamers listed in the database correspond to the raw initial sequences. These aptamers are generally long (80 nucleotides or more) composed of a central random region (typically 20–50 nucleotides) flanked by pre-defined motifs, typically 15–20-nt long, used to hybridize to primers during polymerase chain reaction (PCR). Statistics on all sequences are provided in Supplementary Table S1 and Supplementary Fig. S1. Other aptamers have been optimized and are often (much) shorter: shorter versions of the initial raw sequence are tested, and the shortest active motif is generally selected.

### Analysis of quadruplex propensity

We used the web server iteration [19] of the G4Hunter algorithm [20] to analyze the presence of G4 forming motifs in all sequences. The analysis parameters were set to a length of 25 nucleotides and thresholds of 1.2, 1.5, and 2.0. For aptamers in the database which are <25 nucleotides (the window size selected for G4H), we calculated the G4H for the whole sequence. For example, the 15-nt long TBA GGTTGGTGTGGTTGG would receive a score of 1.133. To confirm the predictions made by G4Hunter, we performed additional analyses with the Quadron algorithm [21] and RNAfold [22] using the “Incorporate G-quadruplex formation into the structure prediction algorithm” option (Supplementary Fig. S2).

### Oligonucleotides and reagents

All sequences were purchased lyophilized from Eurogentec (Belgium) with RP-cartridge purification or polyacrylamide gel electrophoresis (PAGE) purification (for the long sequences) and resuspended in a proper volume of ultrapure water to a final concentration of 100 μM. Sequences are provided in Supplementary Table S4. Concentrations were determined using the extinction coefficients provided by the manufacturer. Biophysical characterizations were performed in buffers containing 10 mM lithium cacodylate which are detailed in experimental sections. All sequences were annealed at 95°C in an appropriate buffer for 5 min and cooled down slowly until reaching room temperature.

### FRET melting competition assay

To confirm G4 formation, we used a combination of techniques, specifically FRET-MC (Förster Resonance Energy Transfer - Melting Competition) which was performed as previously described [23]. Experiments were conducted in a CFX96 Real-Time PCR instrument (Bio-Rad, CA, USA), using 96-well microplates. Each well contained 0.2 μM of F21T fluorescent oligonucleotide and 3 μM of the tested sequence (tested as a competitor) with or without 0.4 μM of PhenDC3, a well-characterized and highly specific G4 ligand. All solutions were diluted in K10 buffer (10 mM lithium cacodylate supplemented with 10 mM KCl and 90 mM LiCl, pH 7.2). Equipment was programmed to record FAM (6-carboxyfluorescein) emission and make an increase of 1°C per minute from 25°C to 95°C. The fluorescent emission curves were normalized using OriginPro2019 software (OriginLab, Massachusetts, USA) to determine the melting temperature (*T*_m_), corresponding to a normalized fluorescence of 0.5. Δ*T*_m_ is calculated by the difference in *T*_m_ of F21T with and without PhenDC3. The S factor was calculated as previously described [23] for each sequence and plotted into a bar graph. Experiments were performed in duplicates on two different plates.

### Fluorescence light-up probe assay

This assay was carried out in 96-well black microplates, and the fluorescence signals were collected using a TECAN Infinite M1000 Pro plate reader (Salzburg, Austria) [24]. ThT (Thioflavin T) and NMM (*N*-methyl mesoporphyrin IX) were excited at 420 and 380 nm, and fluorescence signals were recorded at 490 and 610 nm, respectively. Each well contained 3 μM of pre-folded oligonucleotides and 2 μM of ThT or NMM. All solutions were diluted in K100 buffer (10 mM lithium cacodylate supplemented with 100 mM KCl, pH 7.2), and the plate was shaken for 5 min and incubated for 10 min before reading. This assay was repeated in two independent plates. Positive (G4 forming sequences of different topologies) and negative controls (single-stranded DNA) were used to assess G4 formation. Control experiments in 100 mM LiCl were also performed. Note that the signal is normalized to the number of nucleotides. This means that if a single 20 nucleotide-long G4 is embedded in a long (e.g. 80-nt) raw aptamer sequence, the signal for this long aptamer sequence will appear four times weaker than if the short, 20-nt long version was tested. There are pros and cons with this normalization; yet given the large heterogeneity in oligonucleotide length, we chose to correct the signal for this confounding factor.

### Circular dichroism spectroscopy

To test i-motif or quadruplex formation and stability, we used a Jasco J-1500 CD spectropolarimeter (Jasco, Japan) equipped with a multi-holder and Peltier-type temperature controller. Spectra were recorded between 210 and 330 nm (scanning rate of 100 nm/min, 0.5 nm bandwidth, 1 s integration time over three averaged accumulations), in quartz cuvettes (reference: 115B-10-40 Hellma Analytics, Germany). C-reach sequences were annealed at ∼3 μM in K1 buffer at different pH (10 mM lithium cacodylate, 1 mM KCl, and 99 mM LiCl). G-rich sequences were annealed in K10 buffer. For CD-melting of the C-rich aptamers, ellipticity was recorded at 288 nm as a function of temperature by heating the sample from 25°C to 95°C, with a temperature gradient of 1°C/min.

### UV–vis spectroscopy

The UV–vis experiments were performed on a Cary 300 double-beam spectrophotometer (Agilent Technologies, France) equipped with a thermostable six-cell holder and a high-performance peltier temperature control system (Agilent Technologies, France). The oligonucleotides were diluted to 3 μM and annealed in the same conditions as for CD. Thermal difference spectra (TDS) were used to assess G4 formation by G-rich aptamers (pH 7.2) or i-motifs at different pH. A first spectrum was recorded in conditions favorable for folding (at 20°C), then the temperature was increased to 95°C: at this temperature, most structures are expected to be denatured. The resulting TDS corresponds to the arithmetic difference of the spectra recorded under unfavorable and favorableconditions [25].

## Results

### *I* *n silico* studies

A total of 1495 aptamer sequences were analyzed for G4 propensity. We first used G4Hunter, a simple and widely tool hosted by independent servers in the United Kingdom and the Czech Republic. G4Hunter has been shown to perform well, with its accuracy initially assessed by [20]. Since then, hundreds, if not thousands, of additional sequences were experimentally tested, as shown in [26]. G4Hunter produces very few or no false positives for threshold >1.2 or 1.5, respectively, and its accuracy surpasses that of “pattern” searching algorithms such as Quadparser [20].

**Figure 1.**
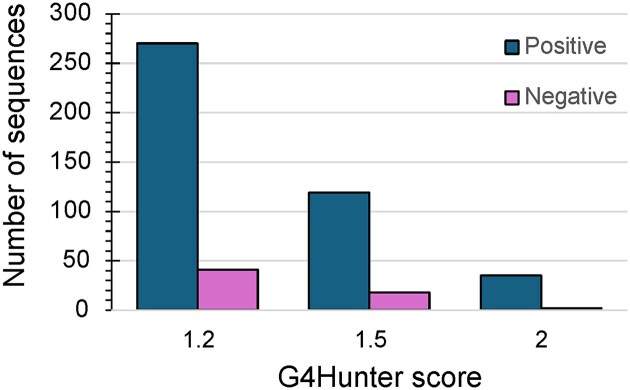
Number of sequences G4Hunter scores with positive and negative values. This figure was generated using data obtained with three different G4Hunter thresholds.

Among these 1495 aptamers, 311 aptamers had sequences with a G4 Hunter absolute score >1.2 (potential quadruplex sequences or PQS), meaning that over 20% of all analyzed aptamers are potentially able to adopt a quadruplex or i-motif fold. G-rich motifs were more frequent than C-rich sequences, with 270 sequences having a positive score (>1.2; G-rich sequences) and only 41 with a negative score (< -1.2; C-rich sequences). Interestingly, G4 formation was only considered in a minority of reports: the term “quadruplex” was only found in 53 cases for these 311 aptamer sequences, meaning that G4 formation was considered in only 17% of cases. Applying more stringent thresholds (1.5 or 2.0) reduces the number of potential G4 candidates, as expected. Specifically, 137 aptamer sequences (9.2%) had a score >1.5, and 37 aptamers (2.5%) had a score >2 (Table 1). The majority of the PQS in the aptamers were found with a positive G4Hunter score across all tested values (Fig. 1).

**Table 1.** Counts of aptamer sequences bearing at least one G4 motif for different G4Hunter scores

G4Hunter threshold	1.2	1.5	2.0
Sequences with G4^a^	311	137	37
Sequences without G4	1184	1358	1458
% of sequences with G4	20.8	9.2	2.5

^a^Some aptamers may bear more than one PQS (there are 365 motifs with a score >1.2).

No algorithm is perfect and most would miss non classical G4-forming motifs. A key limitation of G4Hunter is its accuracy for sequences in the “gray zone,” with scores close to 1.0, which may or may not form G4s. Experimental validation is critical for these sequences. To independently confirm our results, we performed additional analyses using the Quadron algorithm. Quadron is a machine learning-based tool designed to predict the formation of G4s in DNA sequences. It utilizes tree-based gradient boosting machines (GBMs) as its core framework, trained on an extensive experimental dataset of over 700 000 sequences obtained from the human genome using the G4-seq methodology. The results obtained with Quadron confirmed that PQS are abundant in the aptamer database. The Quadron algorithm aligns with the G4Hunter results for a G4Hunter threshold of 1.6. Specifically, Quadron found PQS in 88 aptamers. Among the PQS sequences, 88.6% (78/88) were located on the positive strand and 11.4% (10/88) were located on the negative strands. In other words, the conclusions regarding the biais between G-rich and C-rich sequences are confirmed by two completely independent algorithms for G4 prediction.

We then sought to determine whether these findings could be attributed to chance or if they indicated a genuine enrichment in G4-prone motifs. To do this, we shuffled the nucleotides in the aptamers where G4s had been identified using a G4Hunter score of 1.2 and reanalyzed them. This process aimed to determine if the nucleotides were distributed nonrandomly and if G4s still formed, suggesting that the cause was not simply due to the sequences being relatively rich in guanines. After shuffling, 197 aptamers that previously contained potential G4s no longer did. Only 114 sequences retained at least one G4 after shuffling, meaning nearly 37% of aptamers retained their G4 structure. The total G4 count decreased from 421 to 177, resulting in a loss of 244 G4s, which corresponds to a 58% reduction (Table 2).

**Table 2.** “Shuffled” Aptamers are less likely to adopt a G4 conformation (i.e. have a G4Hunter score >1.2)

Feature	Number		
Sequences analyzed	311		
G4 Count	177		
Sequences with G4	114		
Sequences without G4	195		
Δ sequences with G4 (aptamer seq with G4—shuffled seq)	197	Δ% decrease	36.7
Δ G4 (G4 1.2—shuffled seq G4 count)	244	Δ% decrease	42.0

To confirm the abundance of PQS in the aptamer database we tested the presence of potential quadruplex motifs in randomly generated 80-nt sequences. Using an approach similar to the one used for SELEX, we analyzed 100 000 randomly generated 80-nt sequences and tested for the presence of PQS in these sequences. In order to assess whether the observed difference in the proportion of sequences possessing a specific characteristic in random dataset and in aptamer database selection is statistically significant, we employed a two-proportion *z*-test. The results show that while GQ sequences are statistically significantly over-represented in the aptamer dataset as compared to the random dataset, i-motif sequences are not. This observation remained valid for all G4Hunter scores tested (1.2, 1.5, and 2; Supplementary Fig. S3).

Out of the analyzed aptamer sequences, 912 were DNA and 583 were RNA. Over 24% of all DNA sequences contained G4 structures with a G4Hunter threshold of 1.2, almost 12% at a threshold of 1.5, and over 4% at a threshold of 2.0. The number of sequences containing G4s decreased with higher G4Hunter scores for both DNA and RNA aptamers. Notably, fewer G4 sequences were found in RNA aptamers across all thresholds. Specifically, only 15.4% of RNA sequences had G4s at a threshold of 1.2, and no RNA sequences had G4s at a threshold of 2.0 (Table 3).

**Table 3.** Statistics for DNA and RNA aptamer sequences

Feature	DNA			RNA		
G4Hunter score threshold	1.2	1.5	2	1.2	1.5	2
Sequences with no G4	691	804	875	493	554	583
Sequences with 1+ G4	221	108	37	90	29	0
% of sequences with G4	24.2	11.8	4.1	15.4	5.0	0.0

Finally, taking advantage of an option available on the RNAfold server (http://rna.tbi.univie.ac.at/cgi-bin/RNAWebSuite/RNAfold.cgi), we performed a secondary structure prediction of the long aptamer sequences shown in Supplementary Table S2 to check if these predictions would include G4 folds (this search is relatively limited, as it only looks for “canonical” G4s with four runs of several G, in a “Quadparser-like” mode). RNAfold predicted G4 formation for some (but not all) of the G4Hunter-predicted sequences. Examples are shown in Supplementary Fig. S2. As expected, the G4 forming regions mostly involve guanines in weakly structured (loops or short stems) regions of the RNAfold prediction. In these examples, G4 formation would not disrupt entirely the other secondary structures.

Overall, G4Hunter, Quadron, and RNAfold predicted the presence of stable G4 folds in a number of aptamer sequences. In the next chapter, we experimentally verified thesepredictions.

### Experimental demonstration of G-quadruplex formation

Hundreds of aptamer sequences are prone to G4 formation, according to G4Hunter predictions. Some of these predictions were experimentally confirmed previously, either in the original articles describing the SELEX process, or in follow-up publications (e.g. [27, 28]). Testing all predicted motifs was beyond our means, so we chose to first test motifs with (i) relatively high G4Hunter scores (>1.59) and (ii) no identification of G4 motifs in the original publication. Twelve aptamer sequences against eleven different targets were selected (Table 4). These motifs were between 24- and 40-nucleotide long, with G4Hunter scores between 1.60 and 2.27.

**Table 4.** Sequence of the short G-rich DNA aptamer sequences investigated with high G4Hunter scores—longer versions, corresponding to the full aptamers, are provided in supplementary information (Supplementary Table S2). Sequences are provided in the 5′ to 3′ direction

Name	Sequence	Nt	G4H	Target	Reference
Apt8	TGGTAGGAGGTGCGAAATTGGGGGGGTGGGGTAGCGGT	38	1.61	CDP-ribitol synth.	[49]
Apt22	CGGGAAGAGGGTAAGGGGAGGGAGGGT	27	1.93	A85A prot.	[55]
Apt51	CGGGCAGGGGTGGGGGGGTGTTTGCGGCTCTGGGA	35	1.80	IL2Rα	[56]
Apta14	TGGGCGGGGAGTAGGGAGAGGGGT	24	2.12	HPV	[57]
Apt2 [5]	CGGTGCAGGGGGGGCGGAGAAGAGGTTGAGGGGAGCGGGT	40	1.65	Alloantibody	[58]
Chi46	CGGGGGGGGGAGAAGGCAATGGGGGA	26	2.27	Chitin	[59]
E1	TGGCGGTGGGTGGGGGACAAATTTGGGGGGCGTTGGGT	38	1.79	HepG2	[60]
F1	TGGGTGGTGGGGAGGGGGTTGCTGGGT	27	2.15	HepG2	[60]
FKNS2	CGGGGTGGGTGGGGGGCACGTGTGGGGGCGGCCAGGGT	38	2.00	Fractalkine	[61]
QA12	TGGGTAAGGTCTGGTGGATTGTGGACGGGGGGCGGGGCA	39	1.59	Iridovirus	[50]
RNVL7	CGGCGGGTGGGCGGGGGGAGAACGAGGTAGGGGT	34	1.88	LDLR	[62]
T24	CGGGCGGGGGTGCTGGGGGAATGGAGT	27	1.93	Tetracyclines	[47]

G4H, G4 Hunter score; Nt, length in nucleotides.

**Figure 2.**
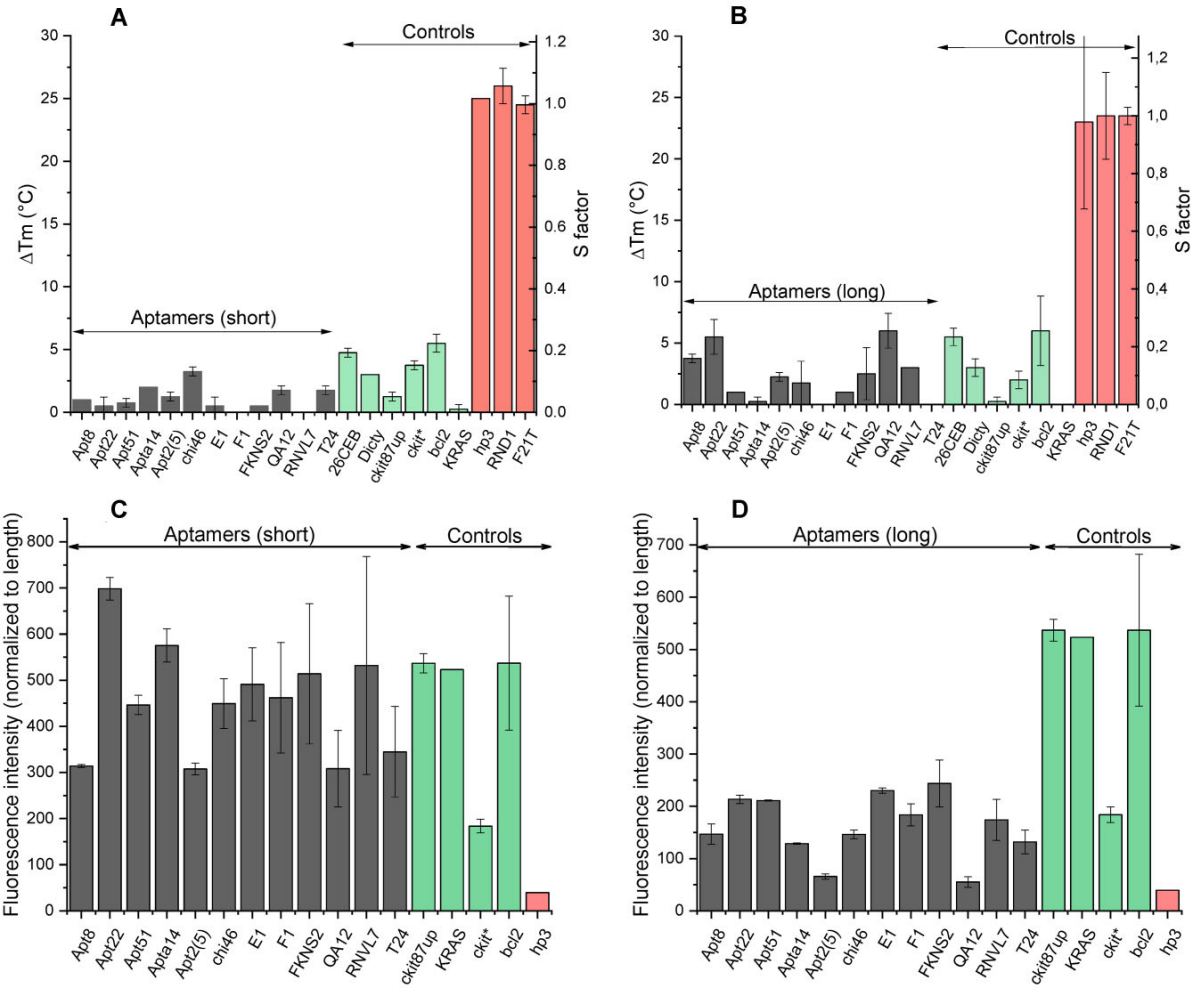
Experimental validation of G4s formation. (**A** and **B**) FRET-MC assay. The bar graph is representing Δ*T*_m_ and S factor for each sequence tested as competitor (3 μM) for the telomeric G4 double labeled F21T (0.2 μM), in presence of a G4 ligand, phenDC3 (0.4 μM). The assay was performed in a 96-well plate in 10 mM licaco supplemented with 10 mM KCl and 90 mM LiCl. (**C** and **D**) Column bar graph plotting NMM fluorescence intensity for each aptamer sequence. Oligos were pre-folded at 3 μM prior fluorescence experiment and incubated with 2 μM of fluorescent light-up probe (ThT data are shown in Supplementary Fig. S5). Errors bars in each panel correspond to the SD calculated from two replicates. Sequences of control oligonucleotides are provided in Supplementary Table S4.

To test G4 formation, we employed a combination of techniques [24]. The FRET-MC assay is a convenient and reliable method to demonstrate G4 formation, provided the structure is thermally stable [23]. As shown in Fig. 2A and B, all tested aptamer motifs (in black) exhibit low Δ*T*_m_ values, similar to those of positive G4-forming controls (in green) and distinct from negative (non-G4-forming) controls (in red).

A second independent assay was then used to confirm G4 formation. NMM becomes highly fluorescent when bound to G4s, particularly those with a parallel conformation [29]. As shown in Fig. 2C, NMM fluorescence in the presence of the short aptamer motifs was comparable to that of the positive controls (in green), confirming G4 formation. This intensity was slightly lower for the full length aptamer sequences (as opposed to just the most G4-prone motifs within each aptamer), but it remained above the level of the negative control in red. Since these intensities were corrected for total length, the results suggest that the full aptamer sequences are long enough to not only adopt a G4 structure but also different folding schemes, as suggested by RNAfold predictions. Little to no increase in fluorescence was observed in the presence of Li^+^, as expected for K^+^ dependent G4 formation (Supplementary Fig. S4).

Additional experiments provided in supplementary information confirm that the tested aptamers form quadruplex structures. Supplementary Fig. S4B and Supplementary Fig. S5 present data obtained using ThT, another fluorescent light-up G4 ligand. A relatively high ThT emission was observed in the presence of all short aptamers, consistent with G4 formation for these sequences. A lower increase was observed in Li^+^, as expected for K^+^-dependent G4 formation. Circular dichroism spectra of all aptamer motifs listed in Table 4 are provided in Supplementary Figs S6 and S7. Circular dichroism is a useful method to investigate G4s, as it not only helps to demonstrate G4 formation but also provides insight into topology [30–32]. All sequences exhibit the CD signature of parallel quadruplexes, with a maximum ellipticity ∼260 nm. Notably, some sequences retain a relatively high CD signal at high temperatures (95°C), as illustrated for Chi46, indicating thermal stability. The topology may depend on the nature of the cation and the presence of flanking nucleotides, as previously reported [33]. Finally, TDS analysis revealed a clear quadruplex signature for all samples, with a negative peak around 295 nm and a positive peak around 273 nm [25] (Supplementary Fig. S8A and B).

Overall, the combination of five independent assays (NMM and ThT light-up effects, FRET-MC, CD, and TDS) confirm G4 formation for all sequences tested in the first series, especially the shorter G-rich motifs.

We next investigated the generability of these findings, and examined a second set of 18 sequences, including 6 RNA aptamers (Table 5). The rationale behind this second series was to test motifs with lower G4Hunter scores, ranging between 1.20 and 1.66. These samples were thus a bit less favorable than the initial 12 candidate motifs tested. We again employed a combination of TDS (Supplementary Figs S8C and D, and S9), FRET-MC (Supplementary Figs S10 and S11), NMM and ThT (Supplementary Figs S4C and D, S12–S15) light-up effects and CD (Supplementary Figs S16 and S17) to study these 18 candidates. Altogether, G4 formation was experimentally confirmed for all sequences with G4Hunter scores >1.31.

**Table 5.** Additional G-rich DNA and RNA aptamer sequences investigated. Sequences are provided in the 5′ to 3′ direction

Name	Sequence	Nt	G4H	Concl
**DNA scores between 1.2 and 1.5**				
0913	GTGTACGGGGTCCGGTAGGGTGGCG	25	1.20	Y(u)
0469	CCTGCAAGCGGGAAGAGGGCAGGGGTGGGAGGGTAACGCGGAAAGGGCA	49	1.20	Y
0053	GTAACCTGTTGGGAGGGCGGGTAGGG	26	1.27	Y
0058	CGGGACGGGTGGGTAAAAGGTGT	23	1.30	^a^
0560	AGGGCTTGGGTTGGGAATAAGGATGTGGGAGGCGG	35	1.34	Y(u)
0143	CCATCTGTGTAAGGGGTAAGGGGTGGGGGTGGGTACGTCT	40	1.42	Y
**DNA scores between 1.5 and 1.8**				
0600	TAGGTGGGTGGGGGACACTGCCCGGGGGTGGTTGGGT	37	1.51	Y
0200	ATGGGGTCGGGCGGGCCGGGTGTC	24	1.54	Y
0646	CTAGGTGGGTGGGGGACACTATCCGGGGGTGGTTGGGTG	39	1.54	Y
0060	TGGGCGGAGGGCTAACGGTGGGGGGATATTATGAGGGGTGGAGGT	45	1.60	Y
0478	CGGCGATGGGGTAGGGGGTGTGGAGGGGCCGGACGGAGGGGCAGCAAGGC	50	1.62	Y
0672	AGGTGGGTGGGGGACACTACTCGGGGGTGGTTGGGTGA	38	1.66	Y
**RNA**				
0902	GGGAUGAGGGACGUGGGAAUCUUGG	25	1.24	Y(u)
1248	GGAAGGAGGGGCAUGCGGUCCAGGG	25	1.28	Y(u)
0898	GGGACCGUGCGAGGGUUGGGUUAGGG	26	1.31	Y
1052	CGGGGGCGUUUCGGCGGAGGAGUGGGA	27	1.44	Y
0861	UGGGGUGUCGGGCGAUUUUUAGGGUUGGG	29	1.48	Y
0192	GGAGAGGGUGGGUGUGCGUGGCGUGGGGU	29	1.55	Y

Nt corresponds to the oligonucleotide length, expressed in nucleotides; G4H, G4 Hunter score. The last column (Concl) summarizes the findings presented in Supplementary Information: Y means that G4 formation is confirmed; Y(u) means that a G4 is formed, but relatively unstable, as shown by FRET-MC.

^a^: Stable G4 formation unlikely based on TDS, CD, and FRET-MC results, even if NMM and ThT fluorescence assays suggest the opposite.

Overall, these results highlight G4Hunter accuracy when utilizing relatively high thresholds: all predictions were experimentally confirmed (no false positive) for sequences with scores >1.31; and all those with a score >1.42 were forming *stable* G4s, as revealed by FRET-MC: this technique only picks thermally stable quadruplexes.

### Investigating i-motif formation

**Figure 3.**
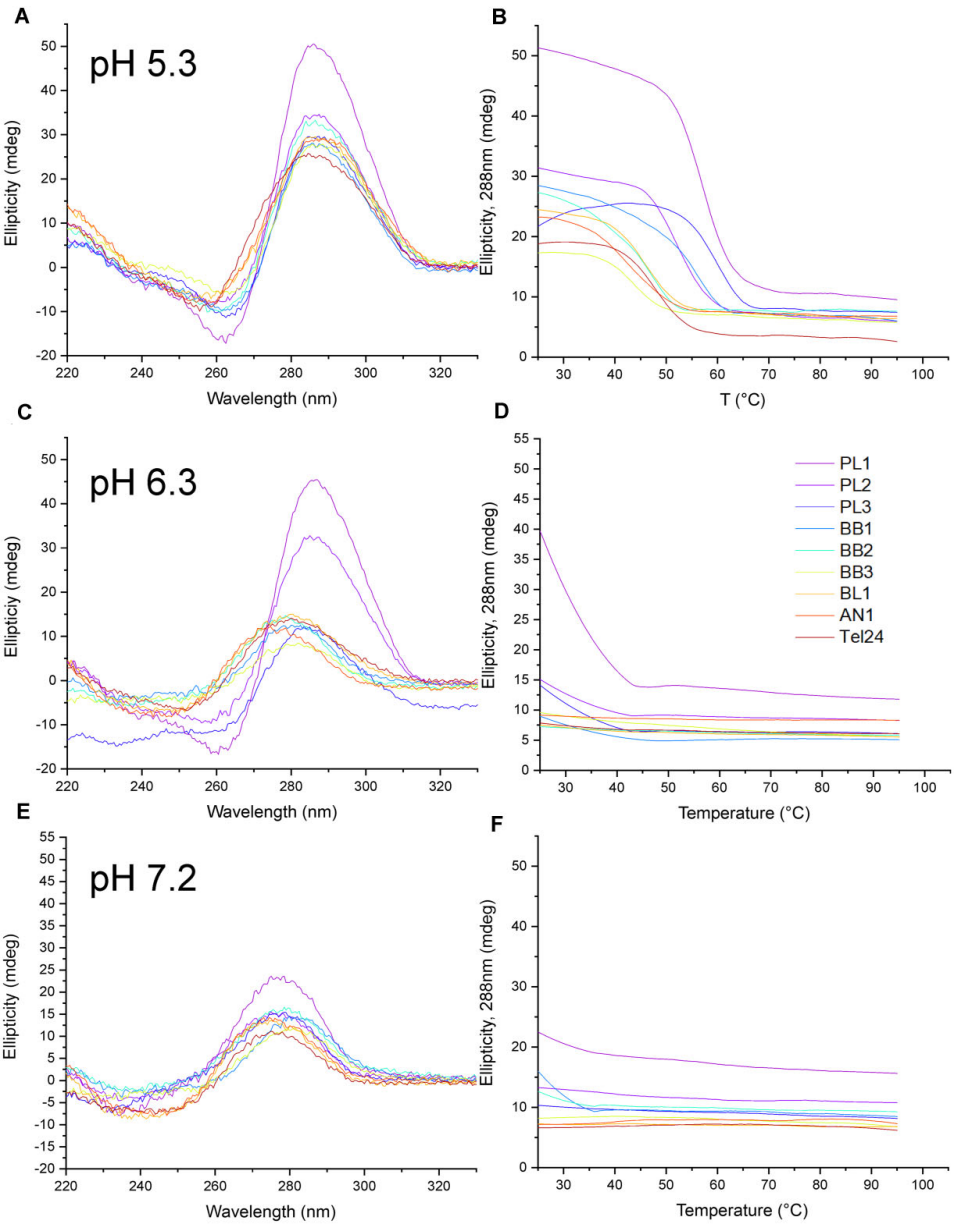
Biophysical characterization of i-motifs. (**A**, **C**, and **E**) Circular dichroism spectra recorded at 25°C at three different pH (respectively 5.3, 6.3, and 7.2) in a 10 mM lithium cacodylate buffer supplemented with 1 mM KCl and 99 mM LiCl. (**B**, **D**, and **F**) CD-melting profiles representing ellipticities at 288 nm as a function of temperature (25–95°C range, 1°C/min, 3 μM strand concentration). Data normalized in Supplementary Information (Supplementary Fig. S18).

Although G4Hunter is not optimized for identifying i-motifs, some sequences with negative G4Hunter scores may still fold into i-motif structures at near neutral pH. Based on our experience with hundreds of C-rich motifs, we have found that highly C-rich motifs are required for stable formation at near neutral pH. A complete list of C-rich aptamers potentially able to form an i-motif is provided in Supplementary Table S3. Among the candidates, eight sequences were selected (Table 6) because they may fold at near neutral pH. These aptamers were identified by four independent studies [34–37]. In the articles where primer sequences were available, we found no obvious bias toward C, indicating that the C-rich motif genuinely resulted from the SELEX process starting from the random central sequence. Three of the four aptamers were raised against proteins, while the last one targeted a small toxin, cylindrospermopsin (Table 6). Next, we searched at which pH the selection was done, and found this information for only three of the four articles: pH 7.4 or 7.5. Even if i-motif formation at this pH is unlikely, we wanted to experimentally measure the stability of the i-motif structure formed by these C-rich DNA sequences. Three pHs were selected: 5.3, 6.3, and 7.2. As shown in Fig. 3A and B (and in Supplementary Fig. S18), all these motifs were able to fold at acidic pH, as shown by their CD spectra, characteristic i-motif TDS signature, and inverted sharp transition at 288 nm upon heating. *T*_m_s were in the 40–60°C range. Unfortunately, stability sharply dropped when increasing pH [38], with only two sequences possibly folded at pH 6.3, and none at physiological pH. Normalized profiles are provided in Supplementary Fig. S18. Finally, TDSs were recorded to confirm i-motif formation (Supplementary Fig. S19). TDS represents the difference in absorbance between unfolded and folded conditions. All profiles recorded at pH 5.3, and some of the profiles recorded at pH 6.2 correspond to the i-motif signature. In contrast, none of the TDS obtained at pH 7.2 match this signature, showing that these sequences were unlikely to adopt an i-motif fold at this pH.

**Table 6.** Sequence of the short C-rich DNA aptamer sequences investigated. Sequences are provided in the 5′ to 3′ direction

Name	Sequence	Nt	G4H	Reference
PL1	CCCCCTCCTCCCTCCCCCACCCGACACTATTCCCCCCCACACC	43	−2.23	[34]
PL2	CCCTCCCCCACCCGACACTATTCCCCCCC	29	−2.31	[34]
PL3	CCCCCTCCTCCCTCCCCCACCC	22	−2.82	[34]
BB1	CCCACCTCGTGATCCCCTTCCCCC	24	−2.00	[35]
BB1	CCTCCCTCCTCATATCCCTGCCCC	24	−1.75	[35]
BB1	CCCGCCCCGCTCCATCCGCCC	21	−1.90	[35]
BI1	CCCACCCACCAGCCCCAACATCATGCCC	28	−1.68	[36]
AN1	CCCTAACAACCAGCCCACCCACCACCCC	28	−1.82	[37]

Nt corresponds to the oligonucleotide length, expressed in nucleotides; G4H, G4 Hunter score (always negative, as it corresponds to C-rich strands).

Overall, given the low stability of these structures at near-neutral pH, it is unlikely that any of these aptamers recognize their targets via i-motif formation, unless ligand-induced folding occurs. In any case, pH-independent alternative folding schemes are possible.

## Discussion

While a number of aptamers are known to adopt a G4 fold, very few have been shown to act via an i-motif structure. An interesting exception was found by Tsvetkov *et al.* who reported an artificial aptamer, called BV42, with a high affinity for hemagglutinin of influenza A [39]. BV42 was proven to contain the i-motif structure even at neutral pH.

In this study, we investigated the G4 and i-motif potential of aptamers found in the UTexas Aptamer Database. The sequences considered in our study span the period 1990–2023. As additional aptamers have been reported since then, there are likely novel G4-prone motifs released since then. In addition, it should be noted that a number of modifications are often introduced in aptamer sequences (e.g. 2′-*O*-methyl or 2′-fluoro). Such modifications were not considered here, as we tested unmodified DNA and RNA motifs. Interestingly, many of these modifications are compatible with quadruplex formation, and some of them actually stabilize G4 structures [40]. Using a combination of prediction tools (G4Hunter, Quadron, and RNAfold), we were able to show that a number of aptamer motifs were prone to form G4s. A comparison of the performance of different prediction algorithms was previously done by Puig-Lombardi *et al.* [41]. G4Hunter and Quadron gave consistent results, and we later experimentally confirmed G4 formation for a number of candidates, using a combination of five methods (ThT, NMM, FRET-MC, TDS, and circular dichroism), as recommended [24]. In contrast, few sequences were predicted to adopt an i-motif fold, and none adopted this conformation at neutral pH. We nevertheless believe that i-DNA constitutes an interesting basis for aptamer selection: it should be interesting to perform SELEX under mildly acidic conditions (6.0–6.5) with a library slightly skewed toward C (>25% C at each position) to favor the identification of i-motif forming aptamers.

On the other hand, the relatively high propensity of aptamer to adopt a G4 fold may explain, at least in part, the slight skewing toward G and C in selected aptamers noted by [5]. RNA aptamers contained on average 30 ± 6.4% G as compared to 24 ± 5.8% C, while DNA aptamerscontained on average 29 ± 8.4% G and 24 ± 6.2% C. This G/C bias may reflect the relatively high frequency of quadruplex-forming aptamers, which must involve a sufficient number of guanines to allow the formation of G-quartets. Note that nucleotide bias may result from other factors: for example, a bias against inclusion of adenine has been reported [42], resulting in aptamers with higher structural stability. PCR and reverse transcription (for RNA aptamers) may also induce other biases [43, 44].

Over 24% of all DNA sequences and 15% of all RNA sequences contained G4 structures with a G4Hunter threshold of 1.2. The higher prevalence of G4-prone DNA aptamers compared to RNA aptamers was initially surprising to us. Several explanations can be proposed: (i) RNA G4s tend to be less polymorphic (often adopting a parallel structure), which may limit their structural variability and complicate the recognition of some epitopes; (ii) G4 RNAs are generally more stable than G4-DNAs. As a result, a lower threshold may be needed to allow stable G4 formation for RNAs, suggesting that we may be underestimating the number of RNA G4-formingaptamers.

In any case, this 24% figure means that nearly one quarter of all listed DNA aptamers possibly (but not necessarily) form G4 structures. A cautionary note shoud be added here: this observation does not mean that 24% do recognize their targets via a quadruplex motif. Even when one can predict that there is a G4 in the aptamer sequence, it does not mean that this constitutes the functional fold for binding, as alternative/competing structure may exist. Other factors may also alter this count. First, the accuracy of our predictions depends on the threshold chosen. Sequences with a G4Hunter score >1.5 nearly always form stable G4s [20, 26]. We confirmed in this paper that 12/12 motifs with scores >1.58 were forming stable G4s, even when embedded in long aptamer motifs. In contrast, sequences with a slightly lower G4Hunter score (between 1.2 and 1.5) are likely to form G4s, but not in a systematic fashion as illustrated in the second set of sequences analyzed. Other very stable secondary structures may also compete with quadruplex formation or form on adjacent regions. These factors may decrease the fraction of G4-forming aptamers. On the other hand, sequences with a score <1.2 (considered here as unlikely candidates) may still form G4 structures. For example, the 15-nt long thrombin binding aptamer (TBA) GGTTGGTGTGGTTGG receives a G4Hunter score of only 1.133, and would therefore be excluded from the list of possible G4s when choosing a threshold of 1.2. Overall, we argue that G4 formation is significantly more frequent than anticipated among aptamer sequences, as we found a number of motifs for which G4 formation was not reported or suspected: out of 311 aptamer sequences containing a potential stable G4 motif, the word “quadruplex” appeared only in 53 of the articles that reported them (17%), despite a high probability of forming stable G4s. Experimentally validating—or invalidating—the role of a G4 core in an aptamer is relatively easy: one can for example mutate G residues potentially involved in G4 formation (using the G4Killer algorithm for example [45]) and determine if these mutants retain their function. Finally, while most of the sequences tested here adopted predominantly intramolecular folds, as shown by nondenaturing electrophoresis (Supplementary Figs S20 and S21), one cannot exclude dimerization or multimerization of the quadruplex core, as found for example for the 93delaptamer [46].

G4-forming functional and useful aptamers exist. These aptamers recognize a variety of targets, including small molecules (e.g. tetracyclins [47] or a known G4 ligand [48]), proteins (e.g. Cytidine Diphosphate (CDP)-ribitol synthase [49]), viruses (e.g. iridovirus [50]), or even nucleic acids (e.g. an L-RNA aptamer recognizing a parallel G4 structure [51]). This demonstrates that G4 motifs may constitute a universal core module for aptamer recognition. One should keep in mind that flanking sequences may affect G4 stability and topology, as shown previously [33]: minimal changes in sequence may have an impact on the G4-based aptamer topology or stability. Attention should therefore be paid to the “trimming” of G-rich sequences during aptamer optimization.

Possible cross-reaction against other G4 binding proteins may be avoided if the 3D fold is actually unique [52, 53]. G4s are actually a family of structures with very different 3D shapes, even if based on the same building block, the G-quartet. There are many possibilities to create diversity, by playing with the number of quartets, bulges, loops, V-shape loops, additional capping interactions, and different molecularities: there are far more different G4 structures than the 3 or 4 known topologies. Yet, the G4 “solution” to the SELEX procedure may become an undesisable one, if this sequence interacts with other G4-binders. Some experts in SELEX actually actively avoid G-rich aptamers, as past experience told them they may lack selectivity. In any case, as this “solution” is often encountered during the SELEX process, we argue that aptamers researchers need to be aware of this possibility: checking for G4 formation makes sense even if (or perhaps, even more if!) this structure is to be avoided. To the very least, one should select the “*Incorporate G-quadruplex formation into the structure prediction algorithm*” option for RNAfold prediction of aptamer motifs, keeping in mind that this software would miss noncanonical G4-forming motifs.

We therefore advocate to systematically assay G4 formation of candidate aptamer sequences whenever a reasonably G-rich motif is selected. Simple methods are actually available to quickly predict and experimentally confirm G4 formation [24]. If G4 structures are to be avoided, one can perform SELEX under potassium-depleted conditions, as this would reduce the stability of the G4. Finally, when the G4 is potentially competing with an alternative fold, this aptamer may actually work as a K^+^/Na^+^ structural switch. We recently studied G/C-rich sequences that can form a quadruplex or a competing hairpin structure based on G-C base pairing, and how the [Na^+^]/[K^+^] ratio influences the structures of these G/C-rich sequences [54]. These motifs were not aptamers though, but correspond to a natural G4 structure with a 9-nt long central loop, in which the loop bases were gradually replaced by cytosines. We identified “shape-shifting” sequences that may respond to [Na^+^]/[K^+^] changes. Quadruplexes, whether to be avoided or sought, should not be overlooked!

## Supplementary Material

gkaf424_Supplemental_File

## Data Availability

All sequences tested are provided in the tables.
