# Supplementary material for: Analysis of quadruplex propensity of aptamer sequences
Source: Nucleic Acids Res. 2025 May 16;53(9):gkaf424. doi: 10.1093/nar/gkaf424 (PMC12082452; doi:10.1093/nar/gkaf424)
Supplement: gkaf424_Supplemental_File [file gkaf424_supplemental_file.docx]

**Analysis of quadruplex propensity of Aptamer sequences**

Anne Cucchiarini^1#^, Michaela Dobrovolná^2,3#^, Václav Brázda^2,3*^, Jean-Louis Mergny^1,2*^

*Supplementary information – Revised MS*

**Supplementary Tables:**

- Length information
- Full (long) sequences of G-rich motifs investigated here
- Complete list of C-rich aptamers potentially able to form an i-motif.
- Control sequences and fluorescent oligonucleotides used

**Supplementary Figures include:**

- Histogram shows the frequency of aptamer length
- RNAfold results
- Aptamers vs random sequences comparison
- Thioflavin T fluorescence assay for G-rich short and long aptamers [1.65<G4H<2.27]
- NMM fluorescence assay for G4-rich aptamers [1.2<G4H<1.8]
- Thioflavin T fluorescence assay for G4-rich aptamers [1.2<G4H<1.8]
- NMM and ThT fluorescence assays controls in lihtium [1.2<G4H<2.27]
- NMM and ThT fluorescence assays of G-rich RNA aptamers in K+/Li+
- CD spectra of G-rich aptamers [1.65<G4H<2.27] between 25 and 95°C
- CD spectra of G-rich aptamers [1.65<G4H<2.27] in Na+
- CD spectra of G-rich aptamers [1.2<G4H<1.8] in K+ and Na+
- CD spectra of G-rich RNA aptamers
- Normalized data of CD melting curves at different pH
- TDS of i-motifs aptamers at different pH
- TDS of G-rich aptamers [1.2<G4H<2.27]
- FRET MC assay of G-rich aptamers [1.2<G4H<1.8]
- FRET MC assay of G-rich RNA aptamers
- Native acrylamide gels [1.2<G4H<2.27]

**Sup. Table S1. Aptamers Lengths statistical summary**

|  | Length of all Aptamers | Length of all Aptamers with G4 1.2 | Length of all Aptamers with G4 1.5 | Length of all Aptamers with G4 2.0 |
| --- | --- | --- | --- | --- |
| Total number of values | 1495 | 311 | 137 | 37 |
| Number of excluded values | 1 | 0 | 0 | 0 |
| Number of binned values | 1494 | 311 | 137 | 37 |
|  |  |  |  |  |
| Minimum | 6 | 6 | 23 | 23 |
| 25% Percentile | 66 | 71 | 75 | 78 |
| Median | 80 | 80 | 81 | 81 |
| 75% Percentile | 88 | 95 | 95 | 88.5 |
| Maximum | 312 | 251 | 251 | 134 |
|  |  |  |  |  |
| Mean | 76.8 | 81.5 | 83.7 | 82.8 |
| Std. Deviation | 24.78 | 25.50 | 27.08 | 17.37 |
| Std. Error of Mean | 0.64 | 1.45 | 2.31 | 2.86 |

**Sup. Table S2. Full aptamer sequences** (5’ => 3’). The parts highlighted in yellow correspond to the G-rich motifs described in the main manuscript.

Apt28L CAGGTCCATCGAGTGGTAGGAGGTGCGAAATTGGGGGGGTGGGGTAGCGGTGTTGAGCACATCGCACTGCTCCTGAACGTAC

Apt22L

GCTGTGTGACTCCTGCAAGCGGGAAGAGGGTAAGGGGAGGGAGGGTAACGCGGAGAAGGCAAGCAGCTGTATCTTGTCTCC

Apt51L

GCTGTGTGACTCCTGCAAATATAGCGGGCAGGGGTGGGGGGGTGTTTGCGGCTCTGGGATCGCAGCTGTATCTTGTCTCC

Apt14L

ATACCAGCTTATTCAATTGGGCGGGGAGTAGGGAGAGGGGTTTCCATCGGCGACAGAGGAGTTATGTGTGTAGATAGTAAGTGCAATCT

Apt2(5)L

AGAGACGGACACAGGATGAGCGGTGCAGGGGGGGCGGAGAAGAGGTTGAGGGGAGCGGGTCCTTCCCCAAGACAGCATCCA

Chi46L

TAGGGAATTCGTCGACGGATCCCCGTAACCCTGCGGGGGGGGGAGAAGGCAATGGGGGACAACTCGCCGGTAGCCATCCATATCTCCAGGTCGACGCATGCGCCG

E1L

CAGCTCAGAAGCTTGATCCTGTGCGATGGCGGTGGGTGGGGGACAAATTTGGGGGGCGTTGGGTGTTTGTGGTGACTCGAAGTCGTGCATCTGCA

F1L

CAGCTCAGAAGCTTGATCCTGTGGGTGGTGGGGAGGGGGTTGCTGGGTCGCGACTAGGAAACTCATGCGGTAAGACTCGAAGTCGTGCATCTGCA

FKNS2L

GTGCAGTCAAAGACGTCCGGGGTGGGTGGGGGGCACGTGTGGGGGCGGCCAGGGTGCTGACCATGAAGTGCGATTGCC

QA12L

GACGCTTACTCAGGTGTGACTCGAGTGGGTAAGGTCTGGTGGATTGTGGACGGGGGGCGGGGCAGTGGCTTGACGAAGGACGCAGAGAAGTCTC

RNVL7L

GGACAGGACCACACCCAGCGCGGTCGGCGGGTGGGCGGGGGGAGAACGAGGTAGGGGTCAGGCTCCTGTGTGTCGCTTTGT

T24L

CGTACGGAATTCGCTAGCGGGCGGGGGTGCTGGGGGAATGGAGTGCTGCGTGCTGCGGGGATCCGAGCTCCACGTG

**Sup. Table S3**. Complete list of C-rich aptamers potentially able to form an i-motif. The most promising regions are highlighted in yellow.

| AGGGCGGCCCCCTCCTCCCTCCCCCACCCGACACTATTCCCCCCCACACCAG | [https://pubmed.nCbi.nlm.nih.gov/**26457419**/](https://pubmed.nCbi.nlm.nih.gov/26457419/) (PLoS 2015) |
| --- | --- |
| CCCTCCCCCACCCGACACTATTCCCCCCCAC | https://pubmed.nCbi.nlm.nih.gov/**26457419**/ |
| GCGGCCCCCTCCTCCCTCCCCCACCCGACACT | https://pubmed.nCbi.nlm.nih.gov/**26457419**/ |
| CCTACCCACCTCGTGATCCCCTTCCCCCAACGT | [https://pubmed.nCbi.nlm.nih.gov/**19545545**/](https://pubmed.nCbi.nlm.nih.gov/19545545/) (BBRC 2009) |
| ACCTCCCTCCTCATATCCCTGCCCCTCTG | https://pubmed.nCbi.nlm.nih.gov/**19545545**/ |
| ACACACCCGCCCCGCTCCATCCGCCCATCG | https://pubmed.nCbi.nlm.nih.gov/**19545545**/ |
| AACACCCCGTCCCATCGACCTCCCTCCTCA | https://pubmed.nCbi.nlm.nih.gov/19545545/ |
| TTCCCCCTCATGACTGCCCTCCCAACG | https://pubmed.nCbi.nlm.nih.gov/19545545/ |
| TGATCCCCTTCCCCCAACGTTGACCTAGA | https://pubmed.nCbi.nlm.nih.gov/19545545/ |
| GTACCCACCCACCAGCCCCAACATCATGCCCA | [https://pubmed.nCbi.nlm.nih.gov/**28006685**/](https://pubmed.nCbi.nlm.nih.gov/28006685/) (Bios 2017) |
| ACCGCAGTACCCACCCACCAGCCCCAA | https://pubmed.nCbi.nlm.nih.gov/28006685/ |
| ACCGCAGTACCCACCCACCAGCCCCAA | https://pubmed.nCbi.nlm.nih.gov/28006685/ |
| GTACCCACCCACCAGCCCCAACATCATGCCCA | https://pubmed.nCbi.nlm.nih.gov/28006685/ |
| CAGTACCCACCCACCAGCCCCAACA | https://pubmed.nCbi.nlm.nih.gov/28006685/ |
| CAGTACCCACCCACCAGCCCCAACA | https://pubmed.nCbi.nlm.nih.gov/28006685/ |
| CCCTAACAACCAGCCCACCCACCACCCCGCCGAGATAG | [https://pubmed.nCbi.nlm.nih.gov/**25122072**/](https://pubmed.nCbi.nlm.nih.gov/25122072/) (Anal 2014) |
| CCCACCCACCAGCCCCGTCAACGACC | https://pubmed.nCbi.nlm.nih.gov/25122072/ |
| CAACCCACCCACCAGCCCCGTCAAC | https://pubmed.nCbi.nlm.nih.gov/25122072/ |
| TACCCACCCGCCAGCCCCAACATCAT | https://pubmed.nCbi.nlm.nih.gov/25122072/ |
| CGAAAAACAUUUCCCCCUCUACCCC | https://pubmed.nCbi.nlm.nih.gov/11590140/ |
| UCCGUACCCAACAUAGAACCCCCCCA | https://pubmed.nCbi.nlm.nih.gov/1697402/ |
| TTCCCCCCCACACCAGTCTTCATCC | https://pubmed.nCbi.nlm.nih.gov/26457419/ |
| CCCCCCCACACCAGTCTTCATCCGC | https://pubmed.nCbi.nlm.nih.gov/26457419/ |
| AAACAUUUCCCCCUCUACCCCAGAC | https://pubmed.nCbi.nlm.nih.gov/11590140/ |
| CAUUUCCCCCUCUACCCCAGACGAC | https://pubmed.nCbi.nlm.nih.gov/11590140/ |
| UUUCCCCCUCUACCCCAGACGACUC | https://pubmed.nCbi.nlm.nih.gov/11590140/ |
| UCCCCCUCUACCCCAGACGACUCGC | https://pubmed.nCbi.nlm.nih.gov/11590140/ |
| ACAAACACCCCCTCGGTTCCACCCCG | https://pubmed.nCbi.nlm.nih.gov/30867884/ |
| ACAAACACCCCCCCGGTTCCATCCCG | https://pubmed.nCbi.nlm.nih.gov/30867884/ |
| AACACCCCCTCGGTTCCACCCCGTCAT | https://pubmed.nCbi.nlm.nih.gov/30867884/ |
| AACACCCCCCCGGTTCCATCCCGTCAT | https://pubmed.nCbi.nlm.nih.gov/30867884/ |
| CUUGUUGACCCCCUCGUUGUCCCCCCCAGACGACU | https://pubmed.nCbi.nlm.nih.gov/9035109/ |
| CCCCCATATCATCAGTCCCACCCCC | https://pubmed.nCbi.nlm.nih.gov/19450981/ |

**Sup. Table S4**. Table of control sequences used in biophysical assays and F21T double labeled nucleotides

| Name | 5’ 🡪 3’ sequence | Comment |
| --- | --- | --- |
| F21T | GGG-TTA-GGG-TTA-GGG-TTA-GGG | (FAM – TAMRA labeling) |
| 26CEB | AAG-GGT-GGG-TGT-AAG-TGT-GGG-TGG-GT | G4-forming positive controls |
| Dicty | GGG-GGA-GGG-GTA-CAG-GGG-TAC-AGG-GG |  |
| Ckit87up | AGGGAGGGCGCTGGGAGGAGGG |  |
| Ckit* | GGCGAGGAGGGGCGTGGCCGGC |  |
| Bcl2 | GGG-CGC-GGG-AGG-AAT-TGG-GCG-GG |  |
| KRAS | AGG-GCG-GTG-TGG-GAA-TAG-GGA-A |  |
| Hp3 | AGGACGGTGTATTTTACACCGTCCT | Hairpin |
| RND1 | CTATACGAAAACCTTTTGTATCATT | Single strand |
| Tel24 | CCCTTACCCTTACCCTTACCCTTA | i-motif |

**Sup. Figure S1**


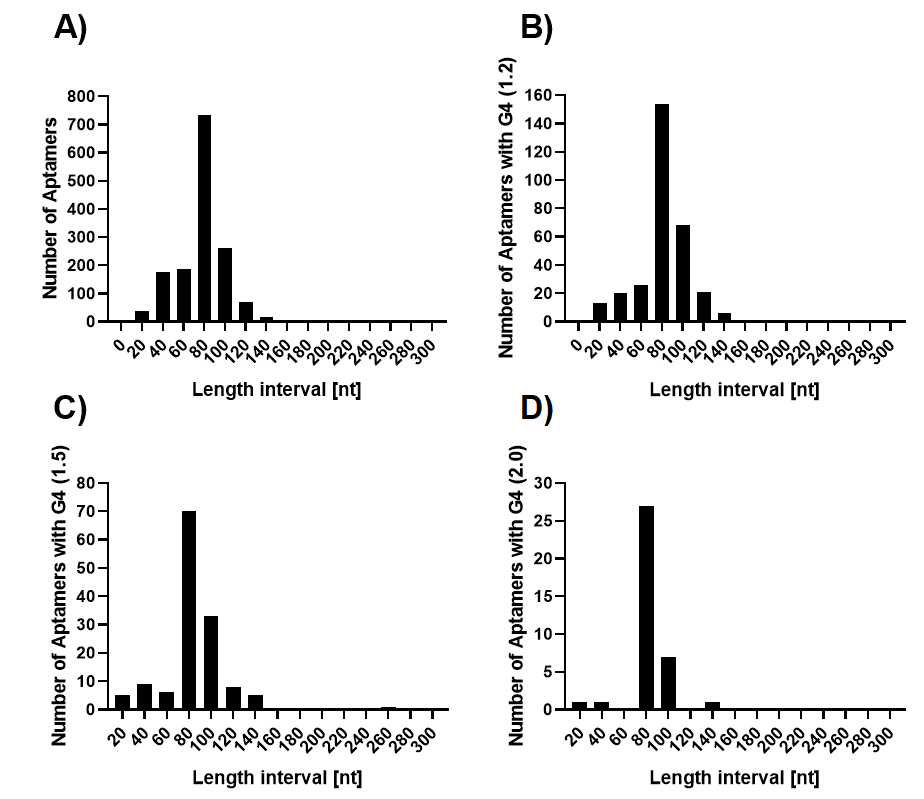


**Sup. Figure S1**. Histogram shows the frequency of aptamer lengths. **A**) all aptamers with a bin width of 20. **B**), **C**), and **D**): size distribution of G4-prone aptamers (containing at least one G4 motif with a G4Hunter threshold above 1.2, 1.5, and 2.0, respectively.

**Sup. Figure S2**


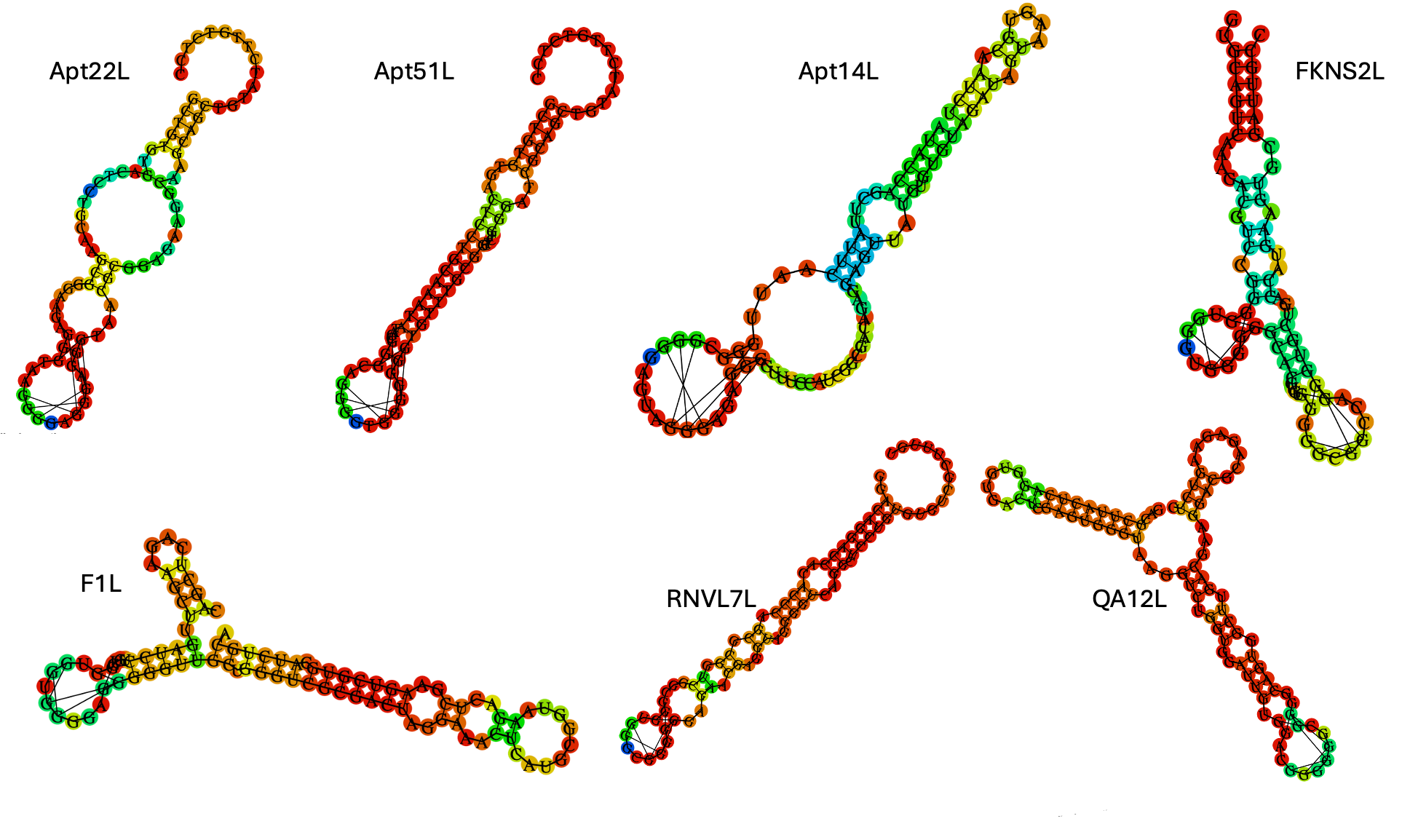


**Sup. Figure S2**: RNAfold **(**<http://rna.tbi.univie.ac.at/cgi-bin/RNAWebSuite/RNAfold.cgi>) analysis of long aptamer sequences using the “*Incorporate G–Quadruplex formation into the structure prediction algorithm*” option. The predicted G4s are depicted by the black solid lines connecting guanines predicted to be involved in G4 formation.

**Sup. Figure S3**


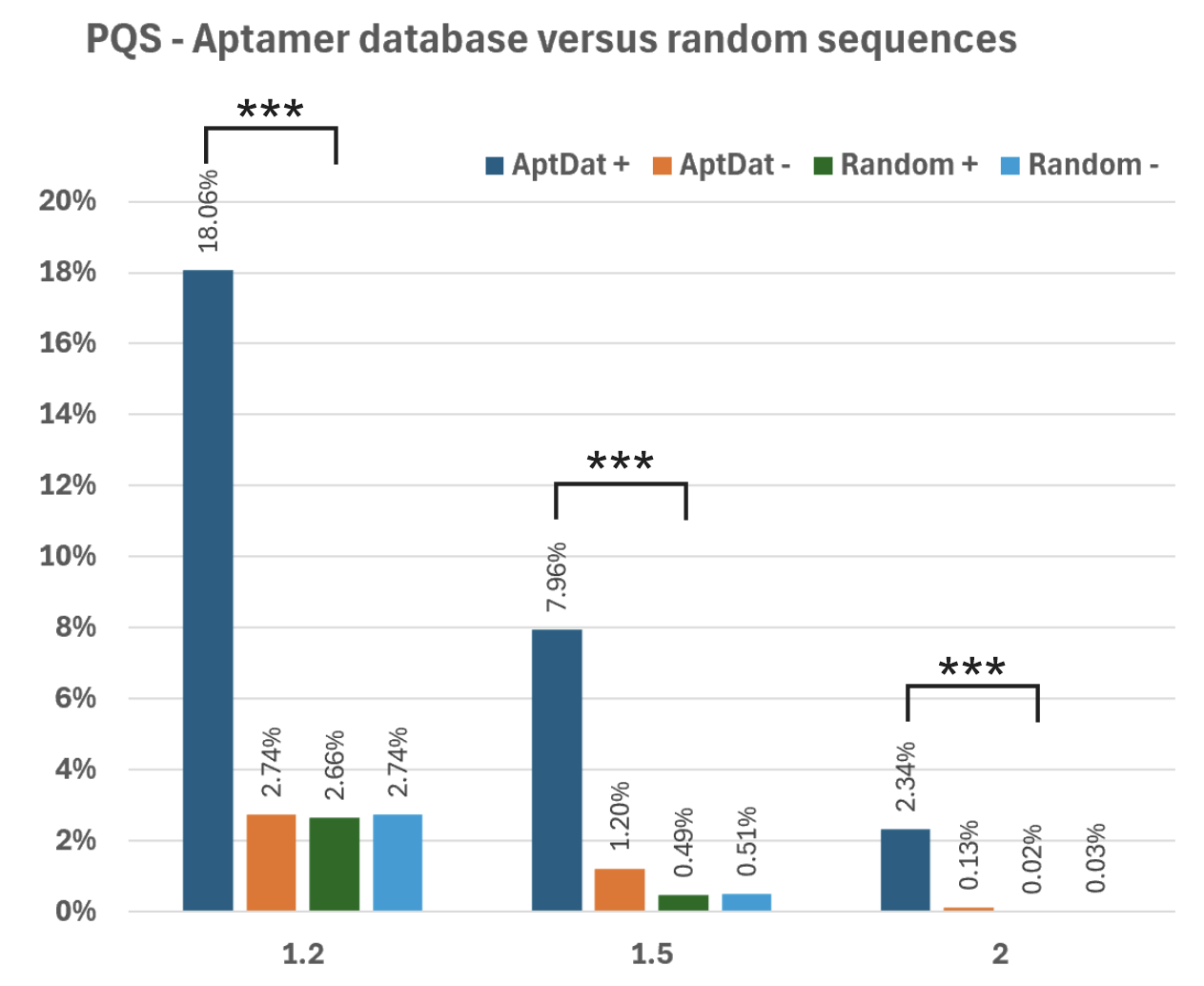


**Sup. Figure S3:** Comparison of PQS percentage in Aptamer database (AptDat) with a random aptamer dataset (Random) on both strains. While the difference between AptDat and Random sequences for G4-forming sequences in the + strain is highly significant, the difference between AptDat and Random sequences for i-motif prone sequences is not significant.

**Sup. Figure S4**

**Sup. Figure S4.** Fluorescence assay controls in Li^+^. Column bar graph plotting NMM (**A**,**C**) and Thioflavin T (ThT) (**B**,**D**) fluorescence intensity for high G4H score aptamer [1.60<G4H<2.27] (**A-B**) and lower G4H score aptamers [1.2<G4H<1.8] (**C-D**). Oligos were pre-folded at 3 µM prior measurments and incubated with 2 µM of ThT. Errors bars in each panel correspond to the S.D. calculated from two replicates. Control sequences are shown on the right; they are either capable of forming a quadruplex in K^+^ (22AG, ckit87up, dicty, 26CEB) or unable to do so (ds26, RND8).

**Sup. Figure S5**

**
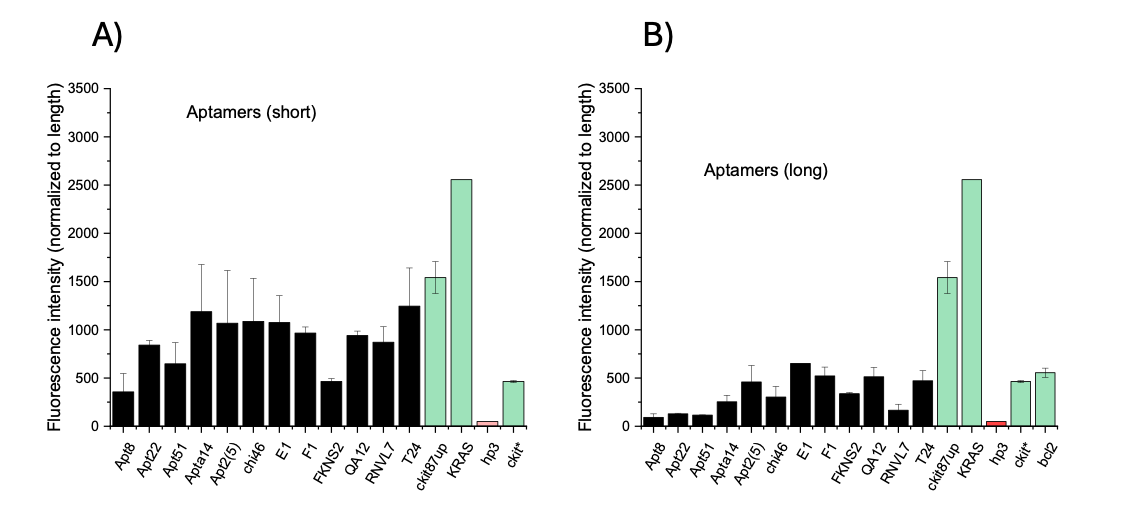
**

**Sup. Figure S5**. Column bar graph plotting Thioflavin T (ThT) fluorescence intensity for each aptamer (either the short or long version). Oligos were pre-folded at 3 µM prior measurments and incubated with 2 µM of ThT. Errors bars in each panel correspond to the S.D. calculated from two replicates. Positive and negative controls (shown in green and red, respectively) are also provided for comparison.

**
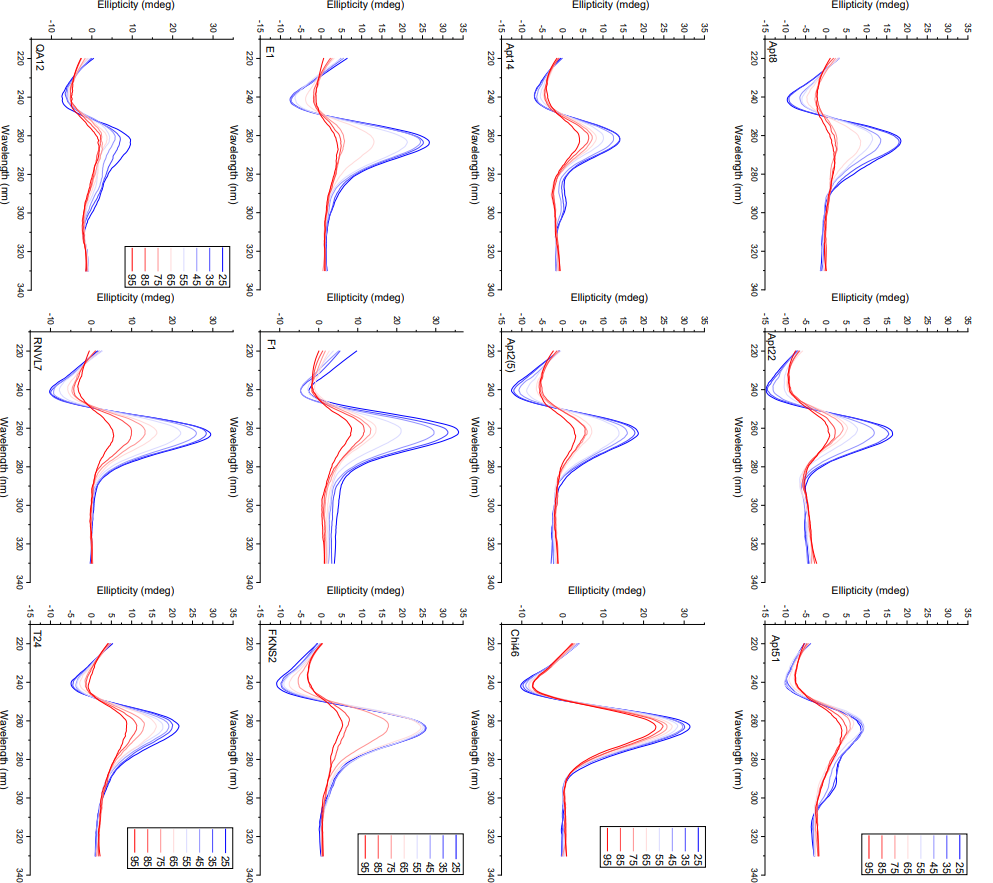
 Sup. Figure S6**

**Sup. Figure S6**. CD spectra of high G4H score [1.60<G4H<2.27] G-rich aptamers (short sequences) at different temperatures. Samples were annealed in K10 buffer and the final concentrations were adjusted to reach an absorbance close to 0.8.

**Sup. Figure S7**

**Sup. Figure S7.** CD spectra of high G4H score [1.60<G4H<2.27] G-rich aptamers at 25°C. Samples were annealed in same buffer (by replacing K+ by Na+) and the final concentrations were adjusted to reach an absorbance close to 0.8, according to Beer-Lambert equation.

**Sup. Figure S8**

**Sup. Figure S8.** TDS profiles of the G-rich aptamers of high G4H score [1.60<G4H<2.27] (**A**-**B**) and lower G4H score [1.2<G4H<1.8] (**C**-**D**). The samples were pre-folded at 3 µM in 10 mM lithium cacodylate, 100 mM KCl before recording a first spectra at 20°C (folded conditions). The second spectra were recorded at 95°C (unfolded conditions). TDS represents the difference in absorbance between unfolded and folded conditions.

**Sup. Figure S9**


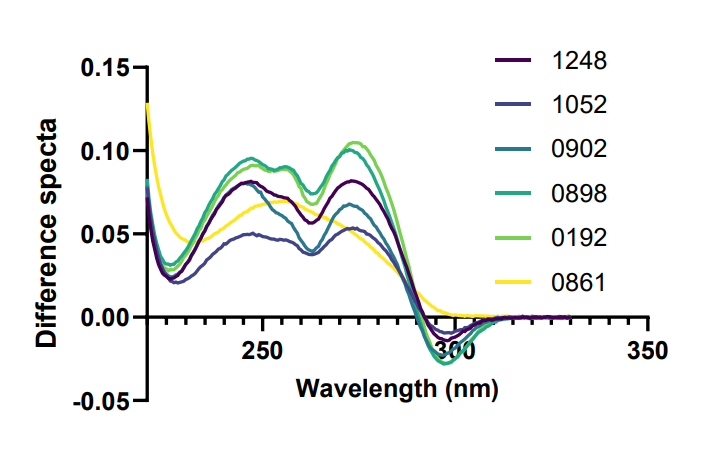


**Sup. Figure S9.** TDS profiles of the G-rich RNA aptamers. The samples were pre-folded at 3 µM in 10 mM lithium cacodylate, 100 mM KCl before recording a first spectra at 10°C (folded conditions). The second spectra were recorded at 95°C (unfolded conditions). TDS represents the difference in absorbance between unfolded and folded conditions.

**Sup. Figure S10**

**
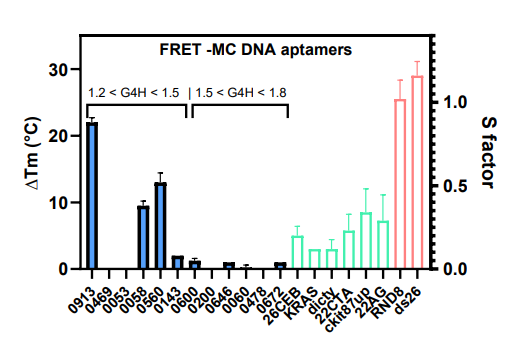
**

**Sup. Figure S10.** FRET-MC assay plotting ∆Tm (also expressed as S factor on the right Y-scale) for each G-rich aptamer [1.2<G4H<1.8]. Positive and negative controls (shown in green and red, respectively) are also provided for comparison.

**Sup. Figure S11**

**
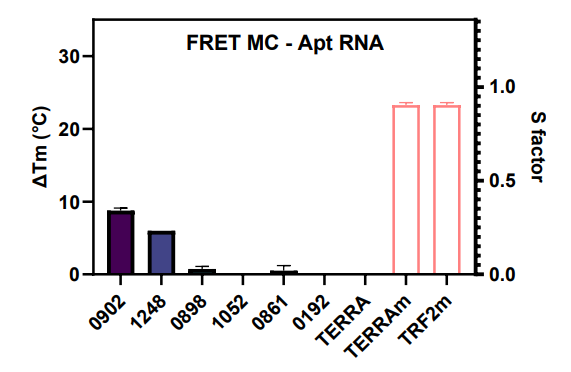
**

**Sup. Figure S11.** FRET-MC assay plotting ∆Tm (also expressed as S factor on the right Y-scale) for each G-rich RNA aptamer. Positive and negative controls are also provided for comparison. TERRAm and TRF2m are mutant sequences unable to adopt a quadruplex fold. TERRA is a positive RNA G4-forming control.

**Sup. Figure S12**

**
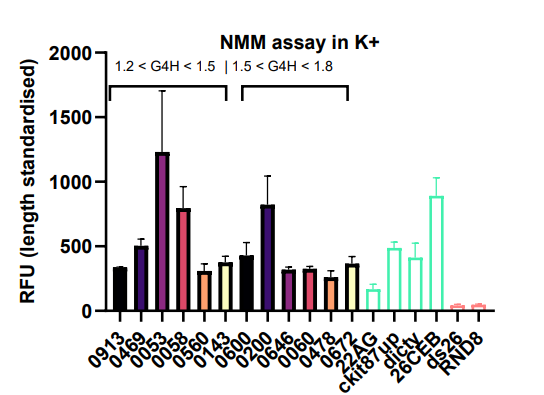
**

**Sup. Figure S12.** Column bar graph plotting NMM fluorescence intensity for each G-rich aptamer [1.2<G4H<1.8]. Oligos were pre-folded at 3 µM prior measurments and incubated with 2 µM of NMM. Errors bars in each panel correspond to the S.D. calculated from two replicates. Positive and negative controls (shown in green and red, respectively) are also provided for comparison.

**Sup. Figure S13**

**
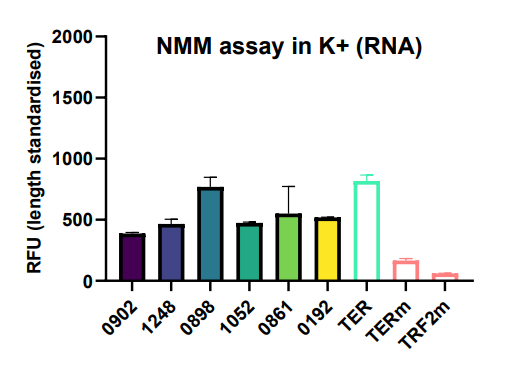
**

**Sup. Figure S13.** Column bar graph plotting NMM fluorescence intensity for each G-rich RNA aptamer. Oligos were pre-folded at 3 µM prior measurments and incubated with 2 µM of NMM. Errors bars in each panel correspond to the S.D. calculated from two replicates. Positive (TER for TERRA, in green) and negative (TERm and TRF2m) controls are also provided for comparison.

**Sup. Figure S14**

**
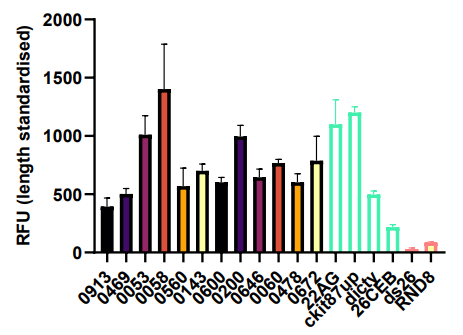
**

**Sup. Figure S14.** Column bar graph plotting Thioflavin T (ThT) fluorescence intensity for each aptamer [1.2<G4H<1.8]. Oligos were pre-folded at 3 µM prior measurments and incubated with 2 µM of ThT. Errors bars in each panel correspond to the S.D. calculated from two replicates. Positive and negative controls are shown on the right, and displayed in green and orange, respectively.

**Sup. Figure S15**

**Sup. Figure S15.** Column bar graph plotting Thioflavin T (ThT) (**A,C**) and NMM (**B,D**) fluorescence intensity G-rich RNA aptamers both in potassium (**A-B**) and lithium (**C-D**) conditions. Oligos were pre-folded at 3 µM prior measurments and incubated with 2 µM of ThT. Errors bars in each panel correspond to the S.D. calculated from two replicates. Positive (TER for TERRA, in green) and negative (TERm and TRF2m) controls are also provided for comparison.

**Sup. Figure S16**

**Sup. Figure S16.** CD spectra of lower G4H score [1.2<G4H<1.8] G-rich aptamers at 25°C. Samples were annealed in potassium buffer (**A-B**) or in sodium (**C-D**) and the final concentrations were adjusted to reach an absorbance close to 0.8.

**Sup. Figure S17**

**Sup. Figure S17.** CD spectra of G-rich RNA aptamers at 4°C. Samples were annealed in K10 and the final concentrations were adjusted to reach an absorbance close to 0.8.

**Sup. Figure S18**

**
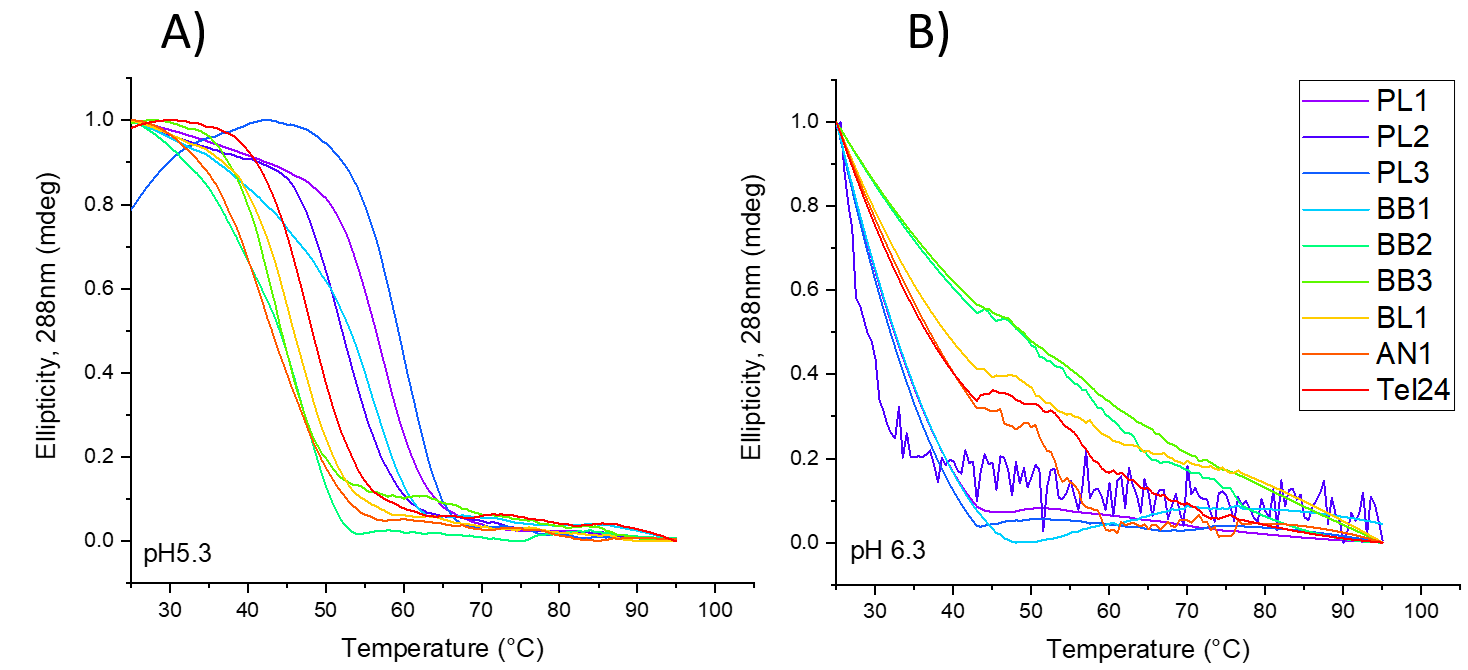
**

**Sup. Figure S18**. Normalized data of the i-motifs aptamers at pH 5.3 (**A**) and 6.3 (**B**) of the CD-melting profiles representing ellipticities at 288 nm as a function of temperature (25 – 95°C range, 1°C/min, 3 µM strand concentration). The raw data were normalized between 0 and 1.

**Sup. Figure S19**

**
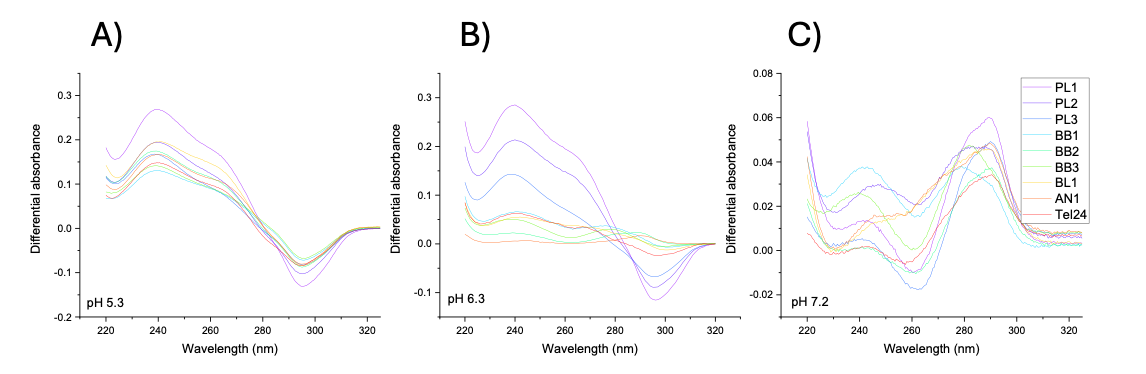
**

**Sup. Figure S19**. TDS (Thermal difference spectra) profiles of the i-motifs aptamers at pH 5.3 (**A**), 6.3 (**B**) and 7.2 (**C**). The samples were pre-folded at 3 µM in 10 mM lithium cacodylate, 1 mM KCl, 99 mM LiCl before recording a first spectra at 20°C (folded conditions). The second spectra were recorded at 95°C (unfolded conditions). TDS represents the difference in absorbance between unfolded and folded conditions.

**Sup. Figure S20**

**
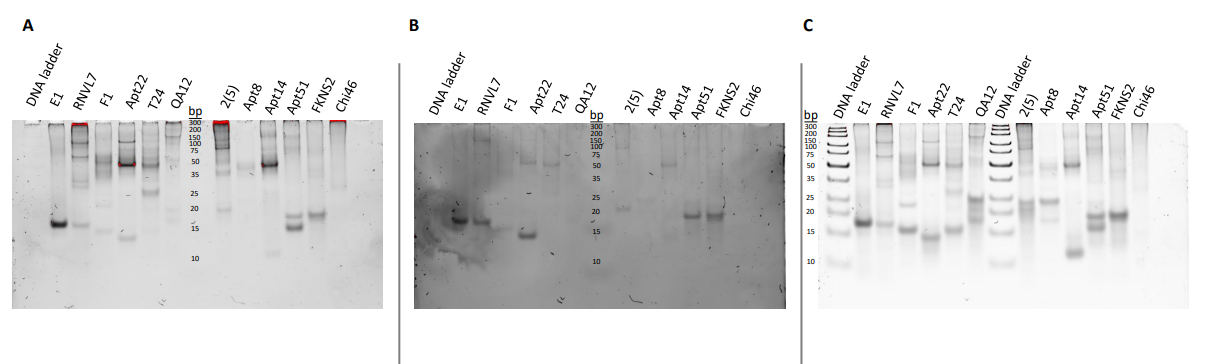
**

**Sup. Figure S20.** Acrylamide (15%) gel of 2µM of the G-rich aptamers [1.60<G4H<2.27], pre-folded in 50mM Tris-HCl, 50mM KCl and colored by ThT (**A**), NMM (**B**) and SYBR gold (**C**). In these experiments, the same gel is revealed with three different methods in succession. SYBR gold is the only staining method that allows the size markers to be visible (G4-specific light up probes will not light up these non-G4 markers).

**Sup. Figure S21**

**
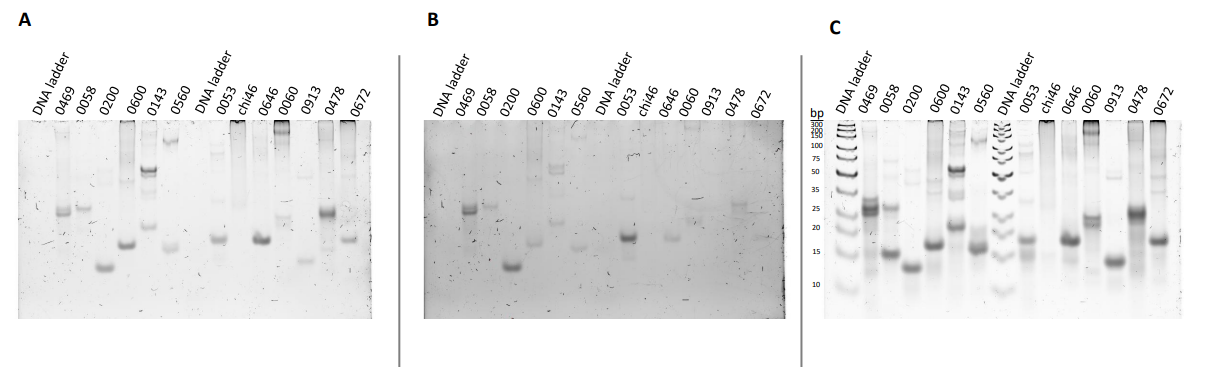
**

**Sup. Figure S21.** Acrylamide (15%) gel of 2µM of the G-rich aptamers [1.20<G4H<1.8], pre-folded in 50mM Tris-HCl, 50mM KCl and colored by ThT (**A**), NMM (**B**) and SYBR gold (**C**). In these experiments, the same gel is revealed with three different methods in succession. SYBR gold is the only staining method that allows the size markers to be visible (G4-specific light up probes will not light up these non-G4 markers).
